# Therapeutic Effects of Natural Products on Human Diseases

**DOI:** 10.3390/life14091166

**Published:** 2024-09-14

**Authors:** Seung-Ho Lee

**Affiliations:** Department of Nano-Bioengineering, Incheon National University, 119 Academy-ro, Incheon 22012, Republic of Korea; seungho@inu.ac.kr; Tel.: +82-31-835-8269

## 1. Introduction

Natural products have long served as potential sources of therapeutic drugs. Numerous pathophysiological conditions, such as inflammation, cancer, viral infection, immunological disorders, and metabolic diseases, have been treated with concoctions or concentrated extracts of natural products [1]. Although numerous drugs have been developed to treat the aforementioned conditions, many diseases still cannot be effectively cured with existing drugs. In addition, discovering new drugs that cause little or no side effects poses significant challenges. Natural products, which are compounds or substances derived from living organisms, could provide answers to these problems because they contain various components that can exhibit unexpected biological properties. Furthermore, individual ingredients derived from natural sources are expected to have synergistic effects when used in traditional medicine [2]. In the last 20 years, approximately one-third of the drugs approved by the Food and Drug Administration have been developed from medicinal plants and their derivates [3]. In addition, the use of various medicinal plant extracts as prescription drugs has gradually expanded in developed countries [4,5,6]. Considering the complex nature of disease progression, drugs containing a single active compound may be ineffective. Natural products for which historical records of therapeutic efficacy are available afford significant advantages in terms of the development of innovative medicines, and recent advances in scientific technologies that can be utilized in profiling analyses of active components, computational prediction, and in vitro/in vivo disease models have reduced the time required to develop innovative drugs based on natural products.

In this Special Issue of *Life* titled, “Therapeutic Effects of Natural Products on Human Diseases”, we have gathered 11 research articles and four reviews that aim to enhance our understanding of the therapeutic effects of various natural products. This Special Issue highlights the previously undiscovered therapeutic effects of natural products against various diseases.

## 2. Highlights from This Special Issue

Vilhelmova-Ilieva et al. evaluated the antiviral potential of Bulgarian propolis extracts. In their study, propolis extracts from Bulgaria were found to be capable of attenuating the attachment and entry of enveloped viruses into host cells, suggesting the potential of Bulgarian propolis extracts for use as antiviral agents [7]. Al-Rajhi et al. found that the chemical composition of laurel leaf extracts (LLEs) can be altered by moist heating. The moist-heated LLEs exhibited enhanced antibiofilm activity. Additionally, the moist-heated LLEs showed higher antioxidant activity and greater inhibitory effects against α-glucosidase and butyrylcholinesterase than LLEs prepared without moist heating. Furthermore, naringenin, a constituent of LLEs, was suggested to serve as a functional component, as the feasibility of molecular docking between naringenin and acetylcholinesterase 1E66 was predicted using computational software, molecular environment 2019 (ver. MOE 2019.0102). These findings suggest that moist-heated LLEs have potential as antibacterial, antioxidant, antidiabetic, and anti-Alzheimer’s disease agents [8]. Interestingly, the chemical constituents of *Lawsonia inermis* extracts decreased with moist heating, resulting in diminished antiyeast and antioxidant properties. These results indicate that the effects of moist heating vary depending on the natural product used [9].

Valadez-Vega et al. conducted an initial exploration of the therapeutic activity of *Bouvardia ternifolia* (Cav.) Schltdl., a plant traditionally used to treat inflammation in Mexico. Different parts of *B. ternifolia* (flowers, leaves, and stems) were extracted using hexane, ethyl acetate, and methanol, respectively. Each extract exhibited varying biological properties in terms of their antioxidant and anticancer activities. These findings suggest that different parts of *B. ternifolia* contain distinct constituents that can be developed as potential therapeutic agents [10].

Park et al. evaluated the antinociceptive effects of *Dendrobii caulis*, which is used as a traditional tonic in China. They found that the oral administration of *D. caulis* effectively attenuated paclitaxel-induced neuropathic pain. Additionally, vicenin-2, which has been identified as a key component of *D. caulis*, can alleviate paclitaxel-induced neuropathic pain by regulating the expression of the transient potential vanilloid 1 (TRPV1) receptor in the spinal cord. These results suggest that *D. caulis* and vicenin-2 could be promising candidates for the development of novel antinociceptive agents [11].

Son et al. investigated the protective effects of Sibjotang, a traditional Korean medicinal formula, against cardiac hypertrophy. They found that administering Sibjotang to an animal model of isoproterenol-induced cardiac hypertrophy effectively reduced the ratio of left ventricular weight to body weight as well as the expression of cardiac hypertrophy markers such as atrial natriuretic peptide and brain natriuretic peptide. These findings suggest the potential of Sibjotang in managing cardiac hypertrophy and its associated heart failure [12].

Lee et al. identified the active compound in *Labisia pumila* leaves through spectroscopic analyses, including nuclear magnetic resonance, infrared spectroscopy (IR), and mass spectrometry. In this study, for the first time, ardisiacrispin A was identified as a functional compound of *L. pumila* leaves that demonstrates anti-lung cancer efficacy [13].

The study by Shaikh et al. showed the inhibitory properties of two flavonoids, eriocitrin and silymarin, on 5α-reductase type II (5αR2) activity. Molecular dynamic simulations between the two flavonoids and 5αR2 provided evidence that both flavonoids have strong interactions with 5αR2. These results suggest the potential of these natural products as strong anti-androgenic alopecia agents [14].

In this Special Issue, two natural products with anti-atopic dermatitis properties are highlighted. Han et al. demonstrated the anti-atopic dermatitis activity of evodiamine, an alkaloid found in *Evodia* fruits [15]. Additionally, magnolol, a major component of *Magnolia officinalis*, was shown to have anti-atopic dermatitis effects by Lee et al. [16]. Both natural products were able to improve atopic dermatitis-like symptoms in 1-chloro-2,4-dinitrobenzene (DNCB)-treated BALB/c mice, suggesting their potential as anti-atopic dermatitis agents.

Cho et al. have presented clinical evidence supporting the reliability of Cuban policosanol. A randomized, placebo-controlled, double-blind clinical study demonstrated that consumption of policosanol (20 mg/day for 12 weeks) effectively protected liver function and improved kidney function. These findings provide valuable insights for the potential use of policosanol in the development of drugs aimed at reducing the toxicity of innovative therapeutics [17].

The review articles cover the biological properties of several natural products and the mechanisms underlying their therapeutic efficacy. Minniti et al. summarized the effects of *Mangifera indica* L. (mango fruit), its by-products, and mangiferin on human health, providing useful information regarding the results of clinical trials on *M. indica* L. or its derivatives [18]. Gwanya et al. highlighted the therapeutic potential of *Bowiea volubilis* subsp. *Volubilis,* commonly known as climbing or sea onion. The authors reviewed the traditional uses of *B. volubilis* and provided insights into its phytochemical composition, bioactive constituents, and therapeutic potential [19]. Phoswa et al. reviewed the effects and underlying mechanisms of *Amaranthus* and *Abelmoschus esculentus* on oxidative stress in the management of diabetes mellitus. The antioxidant properties and therapeutic potential of *Abelmoschus esculentus* summarized in this review may serve as key information for the development of antidiabetic treatments [20].

Finally, Wani et al. reported the therapeutic potential of tangeretin, a polymethoxy flavonoid, against neuroinflammation-induced neurodegenerative disorders. They summarized its effects in various neurodegenerative disease models and provided an overview of its physicochemical properties, pharmacokinetic profile, safety, and toxicity. This review demonstrates the strong potential of using tangeretin as a natural agent to treat neurodegenerative diseases [21].

## 3. Final Reflections

This Special Issue summarizes the biological properties of several natural products and elucidates their therapeutic potential. These findings enhance our understanding of the potential of natural products in meeting the growing demand for safe and effective therapeutics against human diseases.
